# Psychiatrists’ experiences of patient suicide loss: perspectives from residency and supervision

**DOI:** 10.1186/s12909-025-07164-0

**Published:** 2025-05-14

**Authors:** Peri Fenwick, Zainab Furqan, Rachel Beth Cooper, Emmanuel Tse, Andrew Lustig, Mark Sinyor, Arash Nakhost, Paul Kurdyak, David Rudoler, Farooq Naeem, Vicky Stergiopoulos, Juveria Zaheer

**Affiliations:** 1 University of Toronto, Toronto, Canada; 2 Unity Health, Toronto, Canada; 3 University of British Columbia, Vancouver, Canada; 4 Centre for Addiction and Mental Health, Toronto, Canada; 5 Sunnybrook Health Sciences Centre, Toronto, Canada; 6 Ontario Shores Centre for Mental Health Sciences, Oshawa, Canada; 7 McGill University, Montreal, Canada

**Keywords:** Suicide, Qualitative, Patient, Trainees, Supervision

## Abstract

**Background:**

Patient suicide is a common adverse event during psychiatric residency. This study aimed to understand psychiatry residents’ experiences of patient suicide from the perspectives of psychiatrists who experienced this loss as a resident and/or as a psychiatrist supervising residents, and to assess which interventions may help residents feel supported after such tragedies.

**Methods:**

This is a secondary qualitative analysis based on a previous study in which psychiatrists who experienced a patient’s death by suicide were interviewed about their experiences. Of the 18 participants interviewed, 13 participants had experienced the death of a patient by suicide during residency and/or had experience supervising residents in the context of this loss. Direct transcriptions from these 13 interviews were analyzed using constructivist grounded theory.

**Results:**

Participants’ experiences of patient suicide during training were influenced by the practice setting, patient-related factors, learners’ personal circumstances, and the supervisor-trainee relationship. Participants described feeling supported by supervisors from a practical perspective, such as offering a modified workload. Emotional, professional, and existential supports were identified as helpful, though their provision varied depending on the supervisory dynamic. There were differences between resident and supervisor responses to patient suicide, which may be due to residents’ fear of negative evaluations and lack of formal training for supervisors.

**Conclusions:**

The experience of a patient’s death by suicide during residency is diverse and multifactorial. Encouraging connection within the supervisory relationship is critical for both residents and supervisors in coping with the loss and effectively supporting trainees.

**Supplementary Information:**

The online version contains supplementary material available at 10.1186/s12909-025-07164-0.

## Introduction

A systematic review and meta-analysis from 2018 found that 25.7% of individuals who died by suicide had contact with inpatient and/or outpatient mental health services within the year prior to death [1]. Previous research estimates that 33–80% of psychiatrists experience the death of a patient by suicide during their career [2]. Although research on the impact of patient suicide on psychiatrists is limited, a 2023 qualitative study of psychiatrists in Toronto demonstrated that this event is often associated with profound emotional impacts and changes to one’s approach to practice [3].

Experiencing the death of a patient by suicide during training is often associated with impacts on emotional well-being, modifications to clinical practice, and increased concerns about medicolegal issues [4]. A systematic review from 2019 found that the prevalence of experiencing a patient’s death by suicide ranged from 12 to 69% amongst learners during residency, with patient suicide most often occurring during the first year of training [5]. Findings from this study suggest that a patient’s death by suicide impacts trainees more severely compared to senior psychiatrists due to factors such as increased stress from long work hours and sleep deprivation associated with training [5]. Furthermore, 15–39% of participants reported long-term negative consequences after experiencing a patient suicide, ranging from a perceived deficit in their professional abilities to post-traumatic symptoms [5].

Much of the literature on the impact of a patient’s death by suicide on psychiatry trainees has been based on survey data, which has highlighted common emotional responses by residents, such as shock, helplessness, feelings of horror, and anxiety [6]. A systematic review of the impact of patient suicide on mental health practitioners more broadly found that both informal support (e.g., family, friends, and colleagues) and formal support by supervisors were factors that mitigated some of the negative impacts on practitioners [6–8].

Adding to the emerging literature on the impact of patient suicide on trainees, a recent study by Qayyum et al.. examined responses to patient suicide by supervisors [9]. Supervisors reported a lack of training on how to support residents who experience a patient suicide loss [9]. There remains a dearth of investigation in this area. As such, the current study has three main objectives: (1) to understand psychiatry residents’ experiences of a patient’s death by suicide from the perspectives of psychiatrists who experienced this loss as a resident and/or as a psychiatrist supervising residents, (2) to understand which kinds of support are considered most helpful for residents after the death of a patient by suicide, and (3) to explore which interventions may increase the likelihood for residents to feel adequately supported by their supervisors after a patient’s death by suicide.

## Methods

### Data collection

In a previous study, we described the experiences of psychiatrists in Toronto who had a history of providing direct clinical care to a patient who died by suicide [3]. Our current study derives data from this sample. Inclusion criteria include being a current licensed psychiatrist in the Greater Toronto Area who has experienced the death of a patient by suicide during their residency or as a staff psychiatrist. There were no additional exclusion criteria. There were 10 participants initially recruited using email and social media platforms and a snowball sampling strategy was used to recruit 8 additional participants. Informed consent was obtained, and study participants completed a questionnaire which included demographic information and practice characteristics (Appendix A). Using a semi-structured interview guide (Appendix B), individual participants were interviewed for 60–90 min. These interviews began by establishing a background of participants’ personal and professional history, including career trajectory, specialized interests in psychiatry, and day-to-day clinical practice characteristics. Then participants were asked about their experience of a patient’s death by suicide. The interview guide included questions about how the participant was informed about the suicide loss, supports received after the fact, areas for possible improvement, and impacts on future clinical practice. Audio recordings were collected, transcribed verbatim, and de-identified.

### Research design and analysis

This study is presented according to the Standards for Reporting Qualitative Research [10]. The study design and data analysis were based in the principles of constructivist grounded theory, a systematic qualitative research methodology that uses inductive reasoning to derive theory rooted in data [11]. All 17 transcripts were reviewed in-depth by authors ZF, RC, and JZ, with the coding process described in our previous study using NVIVO (qualitative analysis software) to develop a theoretical framework [3]. Coding also focused on experiences of suicide in both residency training and as a supervisor. PF re-read the transcripts and completed (a) secondary coding of the transcripts focusing on experiences in residency and supervision, supervised by JZ, and (b) axial coding with thematic development to identify specific themes based on participants’ experiences during residency and in supervising residents.

This study was approved by the Research Ethics Board (study number 148/2018) at the Centre for Addiction and Mental Health.

## Results

### Characterizing the sample

The sample initially included 18 participants; however, only 17 interviews were available for qualitative analysis due to technical issues with accessing one of the recordings. Of the 17 participants who experienced a patient’s death by suicide as a staff psychiatrist, 13 participants had experienced the death of a patient by suicide during residency training and/or had supervised residents who experienced this loss. Participant demographic and professional information were reported in our previous study [3]. The average age of participants was 46.1 years (range 34–70, SD 11.6) and participants had been in practice for an average of 13.8 years at the time of the interview (range 3–41, SD 12.0).

### Qualitative findings

**Fig. 1:**
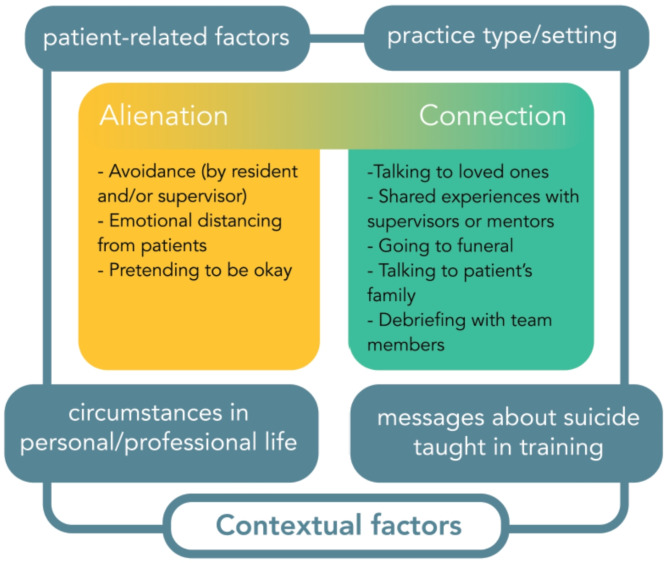
Contextual factors that influence psychiatrists’ experiences of a patient’s death by suicide during residency training

In this section, we present a model for understanding the experience of losing a patient by suicide during residency. This process is conceptualized in Fig. 1. Participant responses to this loss existed on a spectrum between alienation and connection, and there were four main contextual factors that influenced participants’ experiences: (1) patient-related factors, (2) practice type and setting, (3) personal and professional life circumstances, and (4) messages about suicide taught in training. We present a breakdown of each contextual factor using sample quotations from study participants. The perspectives of participants from their role in supervising residents, as well as their recollections from their residency training, are woven together to demonstrate the impact of the supervisor-trainee dynamic in mediating experiences of coping.

#### Patient-related factors

Some participants spoke about feelings of shock, particularly with respect to the unpredictability of patient suicide. For example, certain patients were thought to be improving clinically and were not deemed to be “high risk” for suicide. As per one participant’s recollection from their residency training:

“I think the first suicide that I had, this was in residency…And it was of a person with psychotic depression who I strongly advocated to get ECT and because of the stigma of that treatment, they declined and felt to be capable…I actually accidentally had the opportunity to sit in with my supervisor for their first follow up appointment after the discharge. And that was our last appointment, that they then jumped from a building when their very supportive sister left to get groceries.” *–* Dr. 4.

Another participant highlighted a feeling of fear with respect to the unpredictability of patient suicide:

“Even though I was early in my residency I had that frightening sense that this is not one that I would have predicted.” – Dr. 1.

Although psychiatrists are trained to conduct suicide risk assessments, this clinical skill has an inherent degree of subjectivity and takes time to develop. Patient factors, including the presenting clinical symptoms and psychosocial circumstances, appear to influence the practitioner’s risk assessment and experience of loss.

“It was a young man in university who came in with depression and anxiety… He had come into hospital because he was feeling suicidal and depressed, but he said that resolved completely in hospital… So I remember, I went on vacation. And when I came back I discovered that he had died [by suicide].” – Dr. 1.

#### Practice type and setting

Multiple participants spoke about the unique learning climate of residency and the impact it has on the experience of a patient’s death by suicide. There is a perceived sense of opposing forces during training, such that one may be building a close relationship with patients and simultaneously may feel detached from responsibility due to the hierarchical training structure.

Some participants spoke about the impact of the connection they had with patients during residency, both in brief interactions and in longitudinal care:

“We had met once or twice… as a resident you sort of get attached to clients.” – Dr. 6.

In contrast, for some participants, the transitory nature of clinical interactions within medical training contributed to feelings of detachment from patients who died by suicide. This phenomenon was captured by a participant:

“He wasn’t really my patient, and maybe had he really felt like my patient, I would have had a different reaction.” – Dr. 11.

Acting as the most responsible physician may contribute to an increasing emotional impact on physicians who supervise trainees. As a supervisor, one participant recalled how a trainee seemed unaffected when they learned about their patient’s death by suicide. As a result, the staff psychiatrist reflected on feeling personally responsible for the loss.

“I was taken aback at how [the resident] was…She said, yeah because… it’s all on (you) as (staff physicians).” – Dr. 10.

Meanwhile, from a resident perspective, awareness of not being the most responsible physician may amplify the emotional barrier between the trainee and the impact of the loss.

“I didn’t feel responsible - meaning I wasn’t the primary physician…I really felt so distant from it. In a good way, I guess. I guess it was like, sort of a preparation, trial.” – Dr. 6.

#### Messages taught during training

During residency, there may be a culture whereby patient suicide is viewed as a personal or professional failure, leading to a more reactive stance from learners. One participant recounted this type of response in their role as a supervisor when trying to engage in dialogue with a trainee:

“I could tell when we started to bring it up, that she was like I did everything I could. Like defensive.” – Dr. 6.

Another participant discussed ways in which the training culture can be modified such that the message to residents is not one of shame or failure. They spoke about the importance of normalizing the experience and creating a more supportive narrative:

“I believe residency is an apprenticeship, that the mentors need to be ready to disclose their own experience.” – Dr. 3.

#### Circumstances in personal and professional life

Many individuals pursue a career in psychiatry with a sense of optimism and enthusiasm about working in mental healthcare. One participant identified that experiencing a patient’s death by suicide exposed underlying uncertainties about their chosen career path.

“Feeling that I had missed something. I had some doubts about why I was in psychiatry.” – Dr. 1.

Beyond the daily responsibilities of learning the practice of psychiatry, residency training can be complicated by logistical factors, including increased work-related demands, overnight call shifts and documentation burden. One participant described these competing responsibilities, which can limit the time and space available to process a patient suicide loss and maintain a work-life balance.

“I was distracted by the pragmatics of things needing to be done.” – Dr. 10.

### Encouraging connection: helpful types of support

**Fig. 2:**
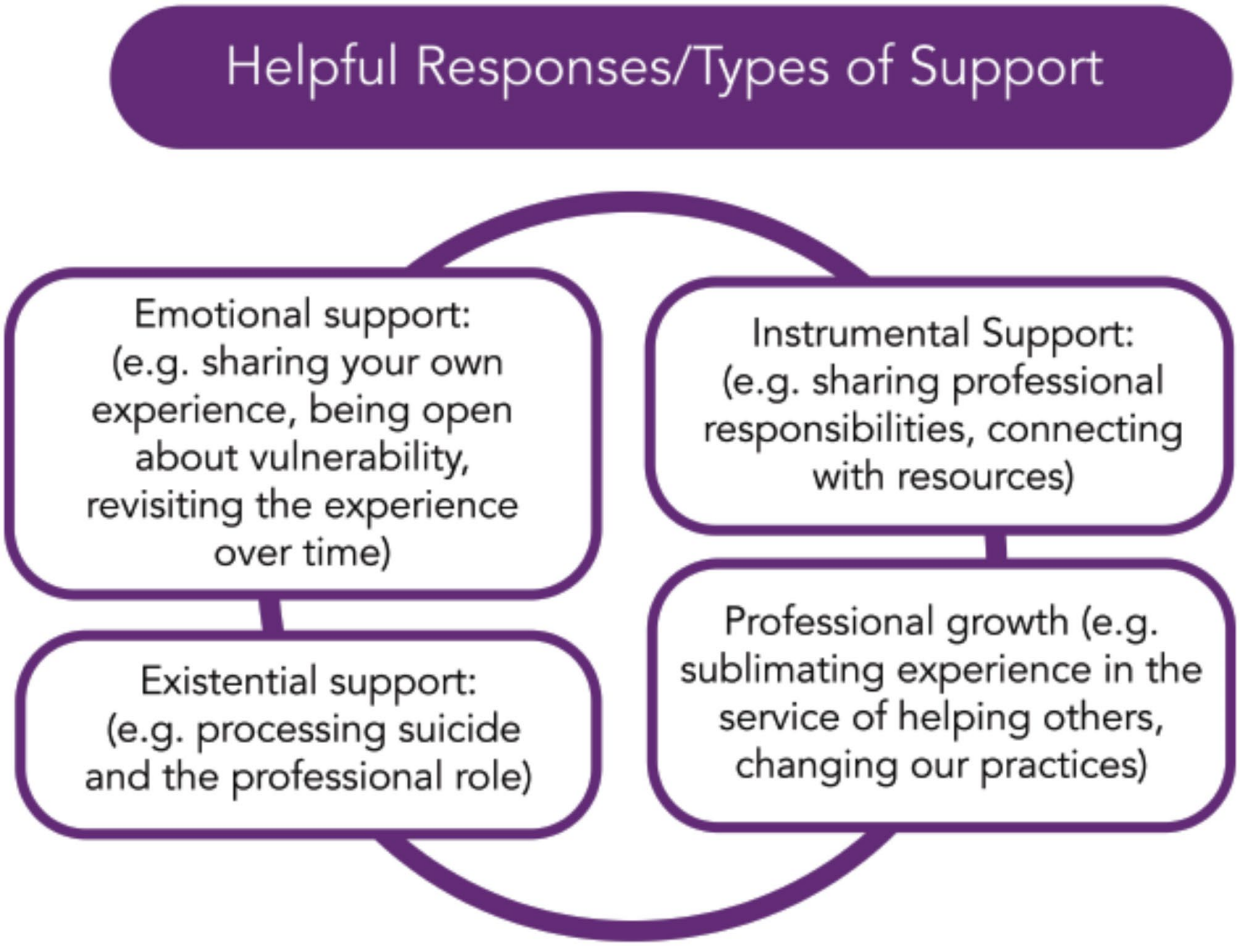
Ways to support learners who experience a patient’s death by suicide

The qualitative data highlighted the importance of supervisor, program, and institutional-level support for residents who have experienced a patient death by suicide. Four main types of support were identified, as depicted in Fig. 2: emotional support, instrumental support, existential support, and professional growth. Underlying these types of support is the foundation of connection to one’s peers, supervisors, and sense of professional identity. We provide a description of the type of support, with quotations from the participants that illustrate each theme.

#### Providing emotional support

Participants highlighted the importance of emotionally supporting trainees who experience the loss of a patient by suicide. Participants spoke about the role of creating space for learners to process the event by normalizing the diversity of reactions.

“I want to just make sure that they understand that many different reactions can happen and it can be difficult and complicated.” – Dr. 6.

In addition to creating an emotionally safe space, another participant described the importance of establishing a confidential, supportive, and judgement-free holding environment for trainees:

“Part of it would be the geography of a safe space. That this is not a hallway conversation, or a cafeteria conversation. It’s a place where people in, sort of a sacrosanct way, can disclose what their deepest worries, doubts and shame are. Without feeling too judged.” – Dr. 3.

Notably, some participants who supervised trainees in the context of a patient suicide were surprised by their trainee’s apathetic reaction to the loss. For example:

“[The resident] didn’t seem that terribly affected by it, curiously…If I were a (resident), I’d be pretty upset about it.” – Dr. 2.

As another participant described:

“The resident wasn’t particularly distressed by [learning about their patient’s death by suicide]…(it was surprising) because they had a longer relationship with this person….I was worried maybe they were having a delayed reaction.” – Dr. 4.

This phenomenon may be related to supervisors’ emphasis on maintaining boundaries in the supervisor-trainee relationship. The impacts appear to be bidirectional, with both parties trying to appear strong and continue work as usual, leading the other to refrain from discussing their experience. One participant spoke about the dynamics of this dyad and its emotional impact:

“It’s like a teacher-learner relationship…It doesn’t feel like it’s equal that I could share how I feel about something versus how they do… I wouldn’t want to upset them or stress them out.” – Dr. 2.

The presence of power differentials was further highlighted by participants, as supervisors described a desire to protect trainees by seeming less emotionally affected by the loss.

“We’re still just forming a relationship in terms of supervisor-student…they don’t want to show vulnerability or they want to present more well because there was a serious outcome and they want to make sure they’re not evaluated poorly.” – Dr. 4.

This phenomenon of emotional distancing may exacerbate feelings of shame and isolation amongst both supervisors and learners. One participant recalled feedback they received from a resident they had previously supervised. The trainee they had worked with expressed feelings of isolation after the death of their patient by suicide due to a perceived lack of support by their supervisor:

“I do realize later, for a while I wasn’t functioning all that well…We still had a good enough relationship that about three, four years later, we met and she said, “I have to tell you, you supervised me really badly on this and I felt very isolated on this death, having to write the discharge summary myself. You were too much out of the picture.” – Dr. 1.

#### Providing instrumental support

Participants described various forms of instrumental support that strengthened a sense of connection in the short-term and long-term after a patient’s death by suicide.

While reflecting on their experience of debriefing with learners after a patient’s suicide, one participant described the role of external support in creating a safe space to be vulnerable and process the experience.

“Just knowing that if there was like an arm’s length person…to give you advice, and you also want advice that’s honest and truthful, right.” – Dr. 7.

The type of support that a trainee may seek and benefit from can vary. One participant recalled a meaningful gesture by their supervisor, which fostered a sense of care and connection:

“My supervisor offered to let me take the day off; she even offered to see all of my outpatients the next week! I didn’t take her up on it but I’ve never forgotten how much she cared.” – Dr. 18.

Similarly, a participant described how their residency supervisor provided reassurance and guidance on how to connect with support in the aftermath of a patient’s death:

“You know, take some time off, we can help you out with that. You know, here are the numbers you should call.” – Dr. 7.

A few participant responses highlighted the role of team-based support. For example:

“The team felt like we did…the best that they could, we had a review with whoever was the clinical lead at the time, and they were very supportive. They set the guidelines, like this is a safe space, and we’re not blaming anybody. We’re trying to improve things, we all want to learn from this.” – Dr. 6.

Participants identified the importance of long-term support and recurrent check-ins. The emotional impact of the event may persist, and establishing avenues for ongoing community and connection during training was perceived as beneficial. As this participant described:

“Make sure [trainees] know who they can go to. Like the counselling services through the university and that even this might come up later for them.” – Dr. 6.

Another participant reflected on the offering of support from a colleague, if needed, in the future:

“He [physician colleague] asked me, ‘do you want to talk about it?’ and I said, no, I’m good, thank you. And he said, just so you know, if you want to talk about it, my door is open… I still appreciated [this gesture] very much.” – Dr. 10.

### Barriers to support

A factor that may contribute to feelings of isolation amongst residents is a perception that supervisors are emotionally distancing themselves from discussing the loss with their trainees. One participant reflected on this experience:

“And I remember even saying to the medical student, “well, is he okay…” and he said, “no, he died!”… And my supervisor later was sort of like, oh yeah, that happened. And he was a bit nonchalant about it.” – Dr. 11.

Similarly, one participant recalled a perceived lack of importance attributed to the event, such that other educational goals were prioritized.

“And I felt a bit shut down, like that hour of supervision could have just been on that topic, whereas we had a topic to review some type of guideline…he maybe could have pulled me in at a different time to let me know about the news, like afterwards, or beforehand.” – Dr. 4.

A possible explanation for why supervisors may avoid discussing the experience with trainees may be related to a lack of training. As one participant described:

“The focus on training residents…but what about…everyone else! How do we help supervisors who are going to need to supervise residents.” – Dr. 5.

Participants expressed a sense of alienation after the critical event, which may be related to an absence of predefined resources available to support physicians. Trainees rely on supervisors for support; however, supervisors may feel ill-equipped due to the absence of top-town resources at the program or institutional level.

“I asked her about what are the institutional responses, either in the residency training program, or in the host hospital, and they’re pretty ill-defined.” – Dr. 3.

Although supervisors may put forth an effort to check in with trainees, if they do not feel adequately trained about how to support residents who experience a patient’s death by suicide, this discomfort may exacerbate feelings of alienation within the resident-supervisor dyad:

“I just want to check in with you [resident], are you okay? But it was a super awkward conversation… I was appreciative that he was calling, but also it was like, I could tell that this was really awkward. And I think he just hadn’t dealt with any resident issues in a very long time.” – Dr. 11.

#### Supporting growth in the context of tragedy: professional and existential support

Participants identified the importance of supporting residents in integrating the experience into the broader context of their professional identity. For example, one physician discussed the role of connecting with others who have been through a similar experience through shared stories:

“I think it would probably be important to talk about… you’re not the only person… this happens. I remember hearing some quotes “so you’re not a real psychiatrist until you’ve had a suicide death.” I don’t know if I agree with that necessarily. But the approach of, this is an outcome, it’s a sad outcome.” – Dr. 5.

Supervisors spoke about providing reassurance to trainees during this challenging time, particularly at times of self-doubt. Mentorship was identified as a prominent form of support when processing the loss of a patient by suicide, as one participant explained:

“Are they going down a path of self-criticism and self-doubt, because what I saw, from the other side, was a lot of them started doubting their clinical acumen, a lot of them were like maybe I’m not cut out to be a psychiatrist. And you need to be there to help these guys.” – Dr. 7.

When there was not sufficient time and space to discuss the experience of losing a patient by suicide in an initial debrief, residents described feeling unsupported and isolated. This phenomenon was captured by a participant who stated,

“I don’t think we ever mentioned the case ever again. It felt strange and unsupportive.” – Dr. 11.

Some participants described a desire to learn from the event and channel the experience into positive changes in patient care.

“Like if I screwed up I want to hear it; I want to make it right.” – Dr. 7.

In contrast to more structured processing through formal debriefs, less structured approaches may be a helpful way to encourage connection and integration of the experience into trainees’ professional growth.

For example, one participant discussed the concept of learning through modelling, particularly with respect to fostering a sense of humanity within psychiatry:

“We went to the funeral and I was given some feedback from some of my mentors that you always go to funerals. So I learned that…you’re never too busy to not value the life. So it is a valued life, and even though it is an expected consequence of working in this field, that you don’t just write it off as just a day in the life of a psychiatrist. Yeah, you honour it…” – Dr. 6.

Supervisors also highlighted the value of informal support. Creating this culture of mentorship within the profession may teach learners to take on similar mentorship roles later in their careers.

“I’ve mentored a lot of medical students, residents and junior faculty. And some of them still come back years later, when they’re at a crossroads or something has happened…I think there’s still a lot of value in the informal ways that we address this…” – Dr. 3.

## Discussion

This study demonstrates that psychiatrists’ experiences of a patient’s death by suicide during training are mediated by multiple factors, including patient-related factors, practice setting, messages taught about suicide during training, and residents’ psychosocial context. Previous research has shown that amongst practicing psychiatrists, feelings of anxiety, guilt, shock, and grief are prevalent in response to patient suicide and mediated by similar elements, including patient, physician, and institutional factors [3]. Although the contextual factors that play a role in the experience of patient suicide loss amongst practicing psychiatrists and residents have notable overlap, the current study highlights unique features related to experiencing a patient suicide loss during training.

Existing literature demonstrates a loss of clinical confidence after the death of a patient by suicide [4, 12]. Similarly, this study underscores a broader impact on feelings of doubt with respect to trainees’ chosen profession. Residents early in their training may be particularly vulnerable to these feelings of doubt as a result of multiple factors, including limited exposure to complex and severely ill patients, the stress associated with adapting to the structure and demands of residency training, and insufficiently developed professional and mentorship networks [12, 13]. This highlights the importance of early, proactive discussion and education about the prevalence of patient suicide in clinical practice, common emotional responses to this loss, and avenues to connect to supports.

Furthermore, psychiatry trainees may experience a higher level of job-related burden and stress compared to other healthcare providers, impacting quality of life and work performance [14]. With the compounding stress associated with losing a patient to suicide, the risk of burnout and physician mental health difficulties increases [15]. Among practicing psychiatrists, stress levels in the weeks following a patient’s death by suicide were shown to be comparable to those seeking treatment after the loss of a parent [16]. Ruskin and colleagues reported increased symptoms of acute stress disorder and post-traumatic stress disorder amongst psychiatric trainees who experienced a patient suicide loss compared to practicing psychiatrists [6]. These vulnerabilities emphasize the need for closer monitoring and implementation of longitudinal supports for residents to prevent more severe long-term mental health impacts.

Finally, the supervisory relationship is critical in how psychiatry residents experience and learn from adverse events during training [17]. Our study demonstrates that there can be mismatches between supervisors’ and trainees’ responses to a patient’s death by suicide. Interestingly, some supervisors perceived a sense of apathy amongst their trainees, which may be interpreted as residents not being significantly impacted by the loss. From a trainee perspective, participants reported supervisor avoidance, or described feeling like their emotional responses to the tragedy were dismissed. Factors that play a role in this mismatch between resident and supervisors’ perceptions include residents trying to appear professional and avoid displays of vulnerability. This may be related to concerns about negative repercussions on their evaluation and progression through training. Some supervisors may feel inadequately trained to have these discussions with learners, or they may be struggling with their own unprocessed emotions. Our findings highlight the importance of open communication about shared experiences and vulnerabilities to help both members of the supervisor-resident dyad cope and grow from such tragedies.

Fortunately, there is growing recognition of the need to support trainees in the context of patient suicide. Efforts include preparing both trainees and supervisors, developing institutional policies, establishing crisis support teams, and implementing postvention protocols [18–20]. While many residency programs provide teaching on suicide risk assessment, a standardized curriculum is lacking [21]. Training mental health professionals in risk assessment has been shown to decrease anxiety and increase perceived confidence in providing care to individuals experiencing suicidality, underscoring its importance in residency education [22]. Postvention programs can also help mitigate the impact of patient suicide on trainees. One such intervention, the SUPPORT program, identifies those most affected by the suicide, facilitates team-based interventions and debriefing, and provides longer-term support [23]. Additional recommendations for supervisors, residency programs, and institutions are listed in Table 1.

Examining the perspectives of psychiatrists who experienced the loss of a patient to suicide at different time points in their careers, as a trainee and as a staff supervisor, adds another layer of understanding related to supervisor-trainee dynamics. Our methodology, which involved a secondary analysis arising from a study of current staff psychiatrists, includes a key limitation such that experiences as a resident and/or supervisor were not an explicit component of the original interview guide and that no current residents were interviewed. This methodological approach requires recollection from a previous time point, which is variable based on the number of years participants have been in practice. Although this may introduce possible recall bias, participants in our study were able to easily recall powerful memories of their experiences during training and their impacts on their personal and professional development.

Exploring the experiences of current psychiatry residents who have experienced the death of a patient by suicide is an essential area for further study. It is important that recommendations such as those listed in Table 1 are implemented and evaluated to determine how to best support trainees and supervisors who experience a patient suicide.

**Table 1:** Considerations for supervisors and institutions in supporting residents

Type of Support	Considerations for Moving Forward
Emotional Support	1. Facilitate training for supervisors on common emotional and behavioural responses from trainees and how to support them after experiencing a patient suicide loss. 2. Create a structured postvention protocol for supervisors and trainees, with an emphasis on proactive communication and creating a safe space to process emotions.
Instrumental Support	1. Encourage top-down training and have resources available for psychiatrists about how to support trainees who have experienced the death of a patient by suicide. 2. Establish supports from a trained practitioner, such as a faculty advisor, to support both psychiatrists and residents around what to expect (institutional and program procedures, timelines, and medico-legal contacts, if indicated) and address challenges that may arise.
Professional and Existential Support	1. Encourage mentorship within the program, both between supervisors and trainees and amongst trainees. Consider creating a support network/program for residents to discuss adverse events with other learners who have been through similar experiences and/or have received specialized training in peer support. 2. Create formal avenues for external support for residents (e.g. psychiatrists not involved in the learner’s residency evaluations) with longitudinal check-ins throughout training.

## Electronic supplementary material

Below is the link to the electronic supplementary material.

Additional File 1: Participant demographic data

Additional File 2: Semi-structured interview guide for qualitative interviews of participants

## Data Availability

The primary data set is not publicly available to maintain confidentiality of the study participants and due to the small sample size and very detailed transcripts.
