# Supplementary material for: Psychiatrists’ experiences of patient suicide loss: perspectives from residency and supervision
Source: BMC Med Educ. 2025 May 14;25:702. doi: 10.1186/s12909-025-07164-0 (PMC12076852; doi:10.1186/s12909-025-07164-0)
Supplement: Supplementary file 1 — Additional File 1: Participant demographic data [file 12909_2025_7164_MOESM1_ESM.pdf]

Qualitative Interview Guide

1. **Venue**: Wherever it is convenient for the participant, and allows sufficient privacy. The location and time will be discussed prior to the meeting.
2. **Duration**: As long as it takes for the participants to complete their stories, although we will try not to go over one hour. If the participant is tired, let him or her take a break. Use your discretion if it is better to go back a second time to continue the interview.
3. **Procedures**:
   - **A. SET UP**
     - **i.** Introduce yourself and the purpose of the interview, e.g., "I am a researcher working on a study of psychiatrists who have experienced a patient suicide in their practice. The purpose of this study is to better understand these experiences and their impact on the clinical practice of psychiatrists."
     - **ii.** Explain the key content in the consent form (e.g., confidentiality and anonymity, the participant's right to withdraw and to delete data).
     - **iii.** Explain the need for audio-recording and obtain approval from the participant. [Remember to bring your recorder and to check it for proper functioning, including sufficient battery life, memory space for recording]
     - **iv.** Obtain written consent.
4. **Open exploration**
   - Start the conversation with a brief prompt, e.g., you may repeat the purpose of the research and invite the participant to share his/her experience, the following are examples of what you may want to say to the participant:
     - o *Thank you for giving us the time to do this interview with you. The main purpose of this interview is to understand your experiences of patient suicide. We are most interested in your personal experience.*

     You can start with whatever you want to talk about first (if participants asked what they should start with).

   - The main purpose of this part of the interview is to allow the participants to express themselves as freely as possible, this can be achieved by keeping in mind that:
     - o *The participant decides what is important to him/her, so let them talk about whatever they want to as much as possible. That means we DO NOT control the agenda rigidly, but try to allow maximum narrative space. You may also want to make sure that you do not interrupt the participant or cut her/him off.*
     - o *Each individual has his/her own idea of what is relevant to the research question. You*

should let them talk even though you may find what he/she says is irrelevant. You may, however, repeat the research question at times to remind.

- *Respect the participant's language by using their expression and their wordings as closely as possible, this will avoid unnecessary (mis)interpretation and narrative conditioning on our part.*
- *Use more prompts and invitations, and use less questions; e.g. invite them to elaborate on or explain about, or give examples for a topic or an experience that they have mentioned. A question-and-answer format tends to put the participant in a passive mode, and severely compromises the opportunity for the participant to volunteer information which is not on your list of questions, therefore defeating the very purpose of ethnographic or discovery-oriented interviewing. If you need to ask questions, ask open-ended and not close-ended questions. Ask specific questions only when you have collected enough information from a topic and need to know the specific details.*
- *Summarize what the participant has said would let him/her know that you've been listening, and help to build a good rapport. This is also helpful when you want to shift the conversation to another topic - make a summary first and smoothly change the topic. Try to be brief with summaries, for long summaries might turn people off.*
- *The purpose of this interview is to explore and discover,* ***NOT*** *to solve problems, provide therapy/counseling, or offer help.*
- *Pay attention to "free information" (content not requires by your question or request, given to you freely): The participant offers as he/she responds to your prompts and questions, these are often things that the participant want to talk more about*

- Please try to jot detail notes during the interview, this will help you to keep track of what has been said and to make summary. Please also note down your impressions, and the participant's non-verbal behaviors whenever possible. These notes can be especially valuable in the unlikely event of recording failure.
- When you think the open exploration part has been completed, try to summarize the main points of the conversation and ask the participant if he/she has any thing more to add. If not, thank him/her for the sharing. Then prepare them for the structured exploration part by saying something like, "In the remaining time, I am going to ask you some further questions."

5. Structured Inquiry

- The purpose of structured inquiry is to focus on specific areas or issues we are interested in, but have not been addressed by the participant in the Open Exploration section. It is hoped that by this time, you would have established a good relationship with the participant and he/she might be

more ready to talk about these topics

- Before we ask the questions, note if any of them had already been answered during the Open Exploration. Ask only those that have not been addressed. Asking the question again will make the participant feel that we have not been paying attention and listening carefully.

Topics of Exploration

- Can you tell me a little bit about yourself? Your background?
- What drew you to psychiatry?
- Can you tell me a bit about your career trajectory since the completion of your residency training?
- What does your day-to-day clinical practice look like?
- Do you have specialized interests within psychiatry?
- How many patient suicides have you experienced during your career, including your years in residency training?
    - Which of these experiences had the greatest impact on you?
- Can you tell me a bit about your connection with the patient prior to the suicide event?
- Can you walk me through the day that you found out about the patient suicide?
- Can you tell me about the days immediately following the event?
    - What did you find most stressful?
    - What did you find most helpful?
- Are there supports that you wish you had received during this time?
- After this event, what was it like for you to see other patients who were experiencing suicidal ideation or behaviours?
- Did you implement any changes to your clinical practice after this event? Can you describe these for me?
- Was there any change in the type of patients that you saw after this event?
- What impact do you think this experience had on you in the long-term? Did this event impact your career decisions moving forward?

Ways in which to ask follow up questions about sensitizing topics (probes and clarification)

1. Can you tell me more about that (person, event)?
2. Can you give me a specific example?
3. Can you explain your answer?
4. In what way?
5. How did you understand that?
6. What does that mean to you?

Wrap up questions

1. Do you have anything to add?
2. Is there anything I should have asked?
3. How did the interview feel for you?
4. Is there anything that surprised you?
5. How are you feeling now?
