# Supplementary material for: Psychiatrists’ experiences of patient suicide loss: perspectives from residency and supervision
Source: BMC Med Educ. 2025 May 14;25:702. doi: 10.1186/s12909-025-07164-0 (PMC12076852; doi:10.1186/s12909-025-07164-0)
Supplement: Supplementary file 2 — Additional File 2: Semi-structured interview guide for qualitative interviews of participants [file 12909_2025_7164_MOESM2_ESM.pdf]

<u>Demographic Form</u>
(Care Provider Version)

Date of Interview: __ __/__ __/__ __ __ __ **(dd/mm/yyyy)**

Study ID: ________________ **Rater Initials:** ___ ___

<u>Please complete the following</u>:

1. **How old are you currently?** ________

2. **What is your gender?** _____

3. **What race do you identify as?**
   - ☐ White
   - ☐ Black or African American
   - ☐ American Indian or Alaska Native
   - ☐ Asian
   - ☐ Native Hawaiian or Other Pacific Islander
   - ☐ Other (please specify)

   ____
   - ☐ Mixed, more than 1 Race
   - ☐ Unknown or Not Reported (participants always have the right to not identify with any category)

4. **When and where did you graduate from medical school?**

5. **Describe your current position**

6. **In a typical week, what % do you devote to outpatient/inpatient/other**

7. **In a typical week, what kind of clients do you see?**

8. **What is your current employment status?**
   - ☐ Full-Time
   - ☐ Part-Time
   - ☐ Self-Employed

9. **What is your funding model?**

10. **Can you describe your last two positions, prior to this one?**

11. **In your recollection, how many clients have you lost to suicide?**
